# Complete Genome Sequence of *Staphylococcus aureus* 6850, a Highly Cytotoxic and Clinically Virulent Methicillin-Sensitive Strain with Distant Relatedness to Prototype Strains

**DOI:** 10.1128/genomeA.00775-13

**Published:** 2013-09-26

**Authors:** Martin Fraunholz, Jörg Bernhardt, Jörg Schuldes, Rolf Daniel, Michael Hecker, Bhanu Sinha

**Affiliations:** Department of Microbiology, University of Würzburg Biocenter, Würzburg, Germanya; Ernst-Moritz-Arndt University, Greifswald, Germanyb; Institute of Microbiology and Genetics, Department of Genomic and Applied Microbiology and Göttingen Genomics Laboratory, Georg-August University, Göttingen, Germanyc; Department of Medical Microbiology and Infection Prevention, University of Groningen, University Medical Center Groningen, Groningen, The Netherlandsd

## Abstract

*Staphylococcus aureus* is a frequent human commensal bacterium and pathogen. Here we report the complete genome sequence of strain 6850 (*spa* type t185; sequence type 50 [ST50]), a highly cytotoxic and clinically virulent methicillin-sensitive strain from a patient with complicated *S. aureus* bacteremia associated with osteomyelitis and septic arthritis.

## GENOME ANNOUNCEMENT

*Staphylococcus aureus* is a Gram-positive human commensal bacterium persistently colonizing the anterior nares of about 30% of the human population. Diverse virulence factors render the bacterium a versatile pathogen that causes a variety of diseases ranging from soft tissue infections to severe conditions (e.g., endocarditis, osteomyelitis, bacteremia, and sepsis). *S. aureus* strain 6850 is a well-characterized prototype strain isolated from a patient with a skin abscess which had progressed to *S. aureus* bacteremia, osteomyelitis, septic arthritis, and multiple systemic abscesses (1). This bacterium is strongly hemolytic on rabbit (2) and sheep blood agar, has a high propensity for cellular invasiveness (3–5), and displays phagosomal escape (5, 6) as well as prominent cytotoxicity (1, 3–5, 7, 8). The strain has been used in a number of studies. Anaerobically grown *S. aureus* 6850 formed minute nonpigmented colonies with reduced hemolytic activity (2). A menadione auxotroph variant, JB1, was generated by a single *in vitro* passage of *S. aureus* 6850 in tryptic soy broth containing gentamicin (2, 9) and has been used to investigate so-called small-colony variants (SCV), noncytotoxic, auxotrophic persister cells (10, 11). A *hemB* mutant of 6850, IIb13 (12), behaving like a stable SCV, has been shown to persist intracellularly and causes less cytotoxicity, resembling the JB1 SCV phenotype (13). Phenotype switching (13), as well as intracellular gene expression in lung epithelial cells (14), has been investigated. *S. aureus* 6850 has also been observed to efficiently escape from endosomes/phagosomes of mammalian cells upon internalization (5). Intravenous infection with strain 6850 resulted in osteomyelitis in a mouse model (15).

Here we report the complete genome sequence of *Staphylococcus aureus* strain 6850. Whole-genome sequencing of the strain was performed by using the 454 GS-FLX system (Roche 454 Life Science, Mannheim, Germany). One 454 shotgun library was generated according to the GS Rapid Library protocol. In total, 254,730 shotgun reads were generated and assembled *de novo* into 45 large contigs (>500 bp) using Roche Newbler assembler software 2.0.00.20 FLX (454 Life Sciences, Roche Applied Science, Branford, CT). PCR-based techniques and Sanger sequencing of the products were used to close remaining gaps. Coding sequences (CDS) were predicted with YACOP (16) using the open reading frame (ORF) finders Glimmer (17), Critica (18), and Z-Curve (19) and were manually curated. tRNAs were predicted with tRNAscan-SE 2.1 (20), and small RNA (sRNA) and rRNA genes were identified by alignment of available sequences. The sequence comprises 2,736,560 nucleotides with a G+C content of 32.78%. The preliminary annotation contains 2,471 ORFs, 57 tRNA genes, and 5 clusters of 16S, 23S, and 5S rRNAs as well as a sixth 5S rRNA locus. *In silico* typing yielded an infrequent *spa* type, t185 (sequence type 50 [ST50], apparently a singleton, not related to common clonal complexes [CCs]) (17; A. Sabat, personal communication). ST50 is reportedly associated with subclinical bovine mastitis (http://saureus.mlst.net) and has been reported to be found both in humans and cattle (21).

### Nucleotide sequence accession number.

The draft genome sequence of *Staphylococcus aureus* strain 6850 is available in GenBank under the accession number CP006706.
